# Neuroleptic Malignant Syndrome: A case report and a literature review

**DOI:** 10.1192/j.eurpsy.2022.1506

**Published:** 2022-09-01

**Authors:** O. De Juan Viladegut, M. Bioque, L. Ilzarbe, H. Andreu Gracia, L. Bueno Sanya, L. Olivier Mayorga

**Affiliations:** Hospital Clínic de Barcelona, Psychiatry And Psychology, Barcelona, Spain

**Keywords:** antipsychotic, Syndrome, malignant, neuroleptic

## Abstract

**Introduction:**

Neuroleptic malignant syndrome (NMS) may be a life-threatening neurologic crisis primarily emerging as an idiosyncratic reaction to antipsychotic agent use, and characterized by a particular clinical syndrome of mental status alter, rigidity, fever and dysautonomia. Mortality results straightforwardly from the dysautonomic manifestations of the disease and from systemic complications.

**Objectives:**

To describe an unusual clinical case in order to determine the management regarding medication and electroconvulsive therapy (ECT), and provide an overview of NMS for the general practitioner with the most up-to-date information on etiology, workup, and management.

**Methods:**

We report a case involving a 55-year-old man with paranoid schizophrenia disorder who presented with hyperthermia, hemodynamic instability, miosis, muscular rigidity, urinary incontinence, catatonic signs and mutism after combining several antipsychotics at the same time: long-acting injectable form of paliperidone, aripiprazol and haloperidol.

**Results:**

Guidelines for specific medical treatments in NMS are based upon case reports and clinical experience. Generally used agents are dantrolene, bromocriptine, and amantadine. A conceivable approach is to start with benzodiazepines along with dantrolene in moderate or severe cases, followed by the addition of bromocriptine or amantadine. ECT is generally reserved for patients not responding to other treatments.

**Conclusions:**

NMS is an uncommon adverse drug reaction, with a multifactor pathophysiology and manifestation. Early diagnosis and interruption of antipsychotic therapy is the first-line treatment, followed by supportive care and pharmacotherapy. ECT is an effective treatment when supportive treatment together with pharmacotherapy fails. It could be considered first line in severe life-threatening situations. It is advisable to consider maintenance ECT due to the high risk of relapse.

**Disclosure:**

No significant relationships.

